# Characterization and Purification of Polydisperse Reconstituted Lipoproteins and Nanolipoprotein Particles

**DOI:** 10.3390/ijms10072958

**Published:** 2009-07-02

**Authors:** Craig D. Blanchette, Brent W. Segelke, Nicholas Fischer, Michele H. Corzett, Edward A. Kuhn, Jenny A. Cappuccio, William Henry Benner, Matthew A. Coleman, Brett A. Chromy, Graham Bench, Paul D. Hoeprich, Todd A. Sulchek

**Affiliations:** 1 Chemistry, Materials, Earth, and Life Sciences; Lawrence Livermore National Laboratory; Livermore, CA 94550, USA; E-Mails: blanchette2@llnl.gov (C.B.); segelke1@llnl.gov (B.S.); fischer29@llnl.gov (N.F.); corzett1@llnl.gov (M.C.); kuhn7@llnl.gov (E.K.); cappuccio2@llnl.gov (J.C.); benner2@llnl.gov (W.B.); coleman16@llnl.gov (M.C.); chromy1@llnl.gov (B.C.); bench1@llnl.gov (G.B.); 2 GWW School of Mechanical Engineering, Georgia Institute of Technology; Atlanta, GA 30332, USA

**Keywords:** apolipoproteins, nanolipoprotein particles, bilayer mimetic, nanobiotechnology, atomic force microscopy, size-exclusion chromatography, lipoprotein crystallization

## Abstract

Heterogeneity is a fact that plagues the characterization and application of many self-assembled biological constructs. The importance of obtaining particle homogeneity in biological assemblies is a critical goal, as bulk analysis tools often require identical species for reliable interpretation of the results—indeed, important tools of analysis such as x-ray diffraction typically require over 90% purity for effectiveness. This issue bears particular importance in the case of lipoproteins. Lipid-binding proteins known as apolipoproteins can self assemble with liposomes to form reconstituted high density lipoproteins (rHDLs) or nanolipoprotein particles (NLPs) when used for biotechnology applications such as the solubilization of membrane proteins. Typically, the apolipoprotein and phospholipids reactants are self assembled and even with careful assembly protocols the product often contains heterogeneous particles. In fact, size polydispersity in rHDLs and NLPs published in the literature are frequently observed, which may confound the accurate use of analytical methods. In this article, we demonstrate a procedure for producing a pure, monodisperse NLP subpopulation from a polydisperse self-assembly using size exclusion chromatography (SEC) coupled with high resolution particle imaging by atomic force microscopy (AFM). In addition, NLPs have been shown to self assemble both in the presence and absence of detergents such as cholate, yet the effects of cholate on NLP polydispersity and separation has not been systematically examined. Therefore, we examined the separation properties of NLPs assembled in both the absence and presence of cholate using SEC and native gel electrophoresis. From this analysis, NLPs prepared with and without cholate showed particles with well defined diameters spanning a similar size range. However, cholate was shown to have a dramatic affect on NLP separation by SEC and native gel electrophoresis. Furthermore, under conditions where different sized NLPs were not sufficiently separated or purified by SEC, AFM was used to deconvolute the elution pattern of different sized NLPs. From this analysis we were able to purify an NLP subpopulation to 90% size homogeneity by taking extremely fine elutions from the SEC. With this purity, we generate high quality NLP crystals that were over 100 μm in size with little precipitate, which could not be obtained utilizing the traditional size exclusion techniques. This purification procedure and the methods for validation are broadly applicable to other lipoprotein particles.

## 1. Introduction

Recently, it has been demonstrated that purified apolipoproteins and liposomes can self-assemble *in vitro* to form discoidal bilayer patches (referred to as nanolipoprotein particles, NLPs) where the lipoproteins are localized to the perimeter [1–12]. These self-assembling membrane mimetics are currently used in biotechnology applications for the solubilization of membrane proteins [13–17]. Important applications of these technology include their use in understanding the function of membrane protein complexes [18] and expressing functional membrane proteins without the use of cells [19]. Despite the potential use of NLPs as a robust model membrane system, the self-assembly process results in particle size variability [2,20,21], where NLP size can span tens of nanometers. Several studies have suggested that the origin of this polydispersity results from different numbers of lipid and apolipoprotein per particle [3,11,22].

Two predominant techniques have been developed for efficient NLP assembly and the major difference between these two techniques is the addition of a detergent, typically cholate, during assembly. In the first method, referred to as cholate-associated assembly, cholate is added to the lipid suspension at a concentration that is above the critical micelle concentration (CMC), resulting in a solution of micelles that consist of cholate and lipid [3,15,16,23,24]. NLPs are then assembled through the addition of an apolipoprotein followed by cholate dialysis. Presumably, the lipoproteins initially associate with the lipid/cholate micelles and upon removal of cholate through dialysis discoidal NLPs form [3,11,12,15,16]. In the second method, referred to as cholate-free assembly, a multilamellar vesicle suspension is tip sonicated to form a lipid suspension of small unilamellar vesicles (SUVs) [11,12,25,26]. This is followed by the addition of apolipoproteins and a 24 hour temperature transition cycle where the reaction temperature is cycled above and below the phase transition temperature of the lipids used for assembly. Presumably the temperature transition cycle promotes defects in the SUVs which allow the lipoproteins to diffuse into the bilayer and repackage lipid into NLPs.

We have recently characterized NLPs assembled from apoE422k (the *N*-terminal 22 kDa fragment of apolipoprotein E4) and DMPC using the cholate-free assembly method by size exclusion chromatography (SEC), native gel electrophoresis, atomic force microscopy (AFM), ion mobility spectroscopy (IMS) and transmission electron microscopy (TEM) [11]. We observed that NLPs assembled in the absence of cholate displayed a single peak by SEC and a single band by native gel, which suggested that the NLPs were monodisperse or homogeneous in size [11]. However, when the particle sizes were examined by AFM, IMS and TEM four discrete NLP sizes were observed indicating that the NLP size distribution was actually highly polydisperse [11]. Although the focus of this work was characterization of NLPs sizes and not purification, the results suggested that utilizing the dominant elution peak during separation by SEC and native gel electrophoresis was not sufficient to separate NLP sub populations under these conditions [11]. This was a surprising result because both of these methods should have the resolving power to separate particles over the size range we observed for E422k-NLPs and these methods have been used to produce monodisperse results and to accurately assess the heterogeneity of NLPs formed with other scaffolds such as human apolipoprotein A1 [15,16,25–28]. Our conclusions from these findings are that some techniques such as SEC or native gel electrophoresis do not separate a subset of heterogeneous lipoprotein samples according to the specification of the separation device; therefore it is important to couple sample separation with high resolution characterization methods. Moreover, other methods for lipoprotein separation, such as field-flow fractionation (FFF) may provide improved fractionation of delicate nanoparticles [29].

We have comparatively examined the separation of different sized NLPs assembled from apoE422k and DMPC in both the absence and presence of cholate by SEC and native gel electrophoresis and report a method to produce homogeneous NLP sub-populations. To assess the importance of NLP monodispersity, heterogeneous and homogeneous NLP sub-populations were subjected to *de novo* crystallization to determine the effect of NLP homogeneity on crystal quality.

## 2. Results and Discussion

### 2.1. Effect of cholate on NLP separation by SEC and native gel electrophoresis

**Figure 1.**
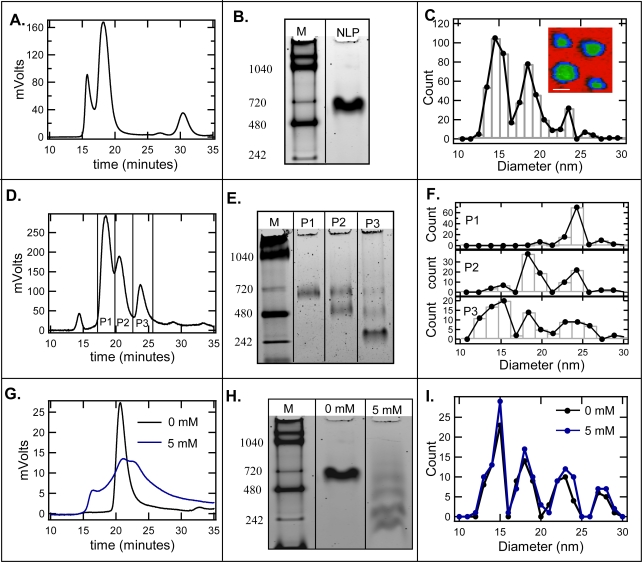
Effect of cholate on NLP size as characterized by SEC, Native gel electrophoresis, and AFM. A. SEC trace, B. Native gel electrophoresis and C. AFM image analysis of NLPs assembled using the cholate-free method. Inset AFM image of the four predominant NLP sizes. Scale bar 25 nm. D. SEC trace, E. Native gel electrophoresis, and F. AFM image analysis of NLPs assembled using the cholate-associated method. G. SEC trace, H. Native gel electrophoresis, and I. AFM image analysis of NLPs assembled using the cholate-associated method in the presence of 0 mM and 5 mM cholate. In G. and I. black line – 0 mM cholate, blue line – 5mM cholate.

When NLPs, assembled using the cholate-free method, were purified by SEC only a singe NLP rich peak was observed (Figure 1A) [6,11,12]. NLP fractions were pooled from the NLP-rich peak and subjected to native gel electrophoresis (Figure 1B). As previously observed, there was a single NLP peak by SEC (Figure 1A) and a single band by native gel electrophoresis (600 kDa) (Figure 1B – lane 2), indicating assembly resulted in homogeneous NLPs that were presumably monodisperse in size. However, multiple sized species with four discrete diameters centered at ~14.5, 19, 23.5 and 28 nm were consistently observed by AFM (Figure 1C). In addition, in previous work we showed that these discrete sizes were not a result of an artifact of AFM imaging, such as tip-sample convolution, and similar discrete sizes were observed with other high resolution techniques such as electron microscopy and ion mobility spectroscopy [11]. It is also worth noting that the majority of NLPs were smaller than 25 nm and the SEC column used in this study (Superdex 200 HR 10/300 column) was expected to efficiently separate these different sized NLP constructs.

To examine the effects of cholate, NLPs assembled using the cholate-associated method were also subjected to SEC purification, native gel electrophoresis and AFM image analysis. In contrast to the results obtained using the cholate-free method, three NLP-rich peaks were observed by SEC (Figure 1D). In addition, when these three peaks (P1, P2, and P3) were subjected to native gel electrophoresis a single band, two bands and three bands were observed in P1, P2, and P3, respectively (Figure 1 E). Unexpectedly, the single band in P1 and the highest MW bands in P2 and P3 migrated to the same MW, (600 kDa), and the lowest molecular weight band in P2 and the middle band in P3 also all migrated to the same MW (500 kDa). In addition, there was a low molecular weight band in P3 (324 kDa) that was not observed in the other two peaks. These banding patterns strongly suggest that these particles represent discrete NLP sizes. When each peak was analyzed by AFM a very similar sizing pattern was observed (Figure 1F). P3 consisted of a single size NLP centered at 23.5 nm in diameter. However, two (19.0 and 23.5 nm) and three (14.5, 19.0, and 23.5 nm) different sized NLPs were observed in P2 and P3, respectively (Figure 1F). The diameter distributions of P1, P2 and P3 corresponded very closely with the banding patterns observed by native gel, where one, two and three different NLP sizes determined by AFM and native gel bands were observed in P1, P2 and P3, respectively (Figure 1E and 1F). Interestingly, these discrete sizes were centered at the same means observed for the NLPs assembled in the absence of cholate and there was no dramatic change in particle shape, which suggests that cholate does not appreciably affect the overall structure of the NLPs.

To assess whether the addition of cholate after cholate-free NLP assembly can promote separation by SEC and native gel, 5 mM cholate (below the CMC of cholate) was added to NLPs that were assembled in the absence of cholate. Figures 1G and 1H are the SEC and native gels, respectively, of the NLPs before and after cholate addition. As shown in Figure 1D, the single NLP peak became more broad displaying two overlapping peaks after the addition of 5 mM cholate. In addition, the single band in the native gel became four bands, which closely corresponded to the diameter distribution determined by AFM. To determine the effect of the post-addition of cholate on the NLP diameter distributions, the sample was imaged both before and after cholate addition (Figure 1I). From this analysis no changes were observed in either the mean or relative fraction of each discrete size (Figure 1I). It is also worth noting that the emergence of a small lipid-rich peak in the SEC trace after cholate addition (Figure 1D) was observed, suggesting that a small fraction of the particles have started to disassemble. This was expected since detergents will also promote the formation of micelle/lipoprotein complexes [24]. However, we did not observe any micelle-like structures by AFM, indicating that the constructs in the lipid-rich region most likely do not have favorable interactions with the mica surface under the buffering conditions used.

These combined results strongly suggest that even though the NLPs were dialyzed after cholate-NLP assembly a portion of the cholate may remain associated with the NLP construct. This is not surprising since it is known that the amphiphilic nature of cholate will cause them to intercalate into the NLP bilayer, and removal through dialysis alone may not be sufficient to remove all of cholate that is anchored into the NLP bilayer. The most intriguing aspect of these results is the effect cholate has on NLP separation by SEC and native gel. These results suggest that NLPs may be interacting with one another during electrophoresis and SEC purification such that different sized particles migrate to the same spot on native gel (~ 620 kDa) and elute with similar retention times during SEC purification. Quite possibly, these interactions may be disrupted when negatively charged cholate becomes associated with the NLPs, allowing for improved separation during SEC and native gel electrophoresis. It is worth noting that cholate appears to improve NLP separation with SEC, however both P2 and P3 still contained particles with a variety of diameters and thus are still polydisperse. In addition, the fact that no change was observed after cholate addition indicates cholate does not appreciably affect the absolute size of these particles.

### 2.2. De-convolution of the single NLP peak

The observation that NLPs of different sizes all eluted in a single SEC peak when NLPs were assembled using the cholate-free method strongly suggest that the particles are interacting dynamically, but it is still unclear whether the elution pattern of these particles is consistent across the entire peak. Therefore, the elution properties of the four different NLP sizes in the single NLP rich SEC peak were resolved by collecting 27 fractions across the entire peak and subjecting select fractions to AFM image analysis. In these experiments, two 20 mg NLP assemblies were pooled so there was sufficient material in each fraction for subsequent analysis.

**Figure 2.**
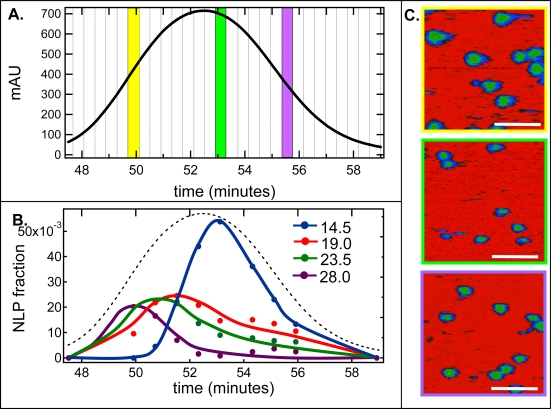
AFM analysis of SEC fine elution fractionation vs. volume purification. A. An expanded view of the NLP-rich peak under cholate-free assembly conditions. Dashed lines indicate the fractions in which elutions were collected. B. Relative proportion of the four different sized NLPs in the SEC fine elution fractions. Dashed line represents SEC trace. C. Representative AFM images from the fractions highlighted in A., highlighted color of the fraction correspond to the images with the same colored border. Scale bar 100 nm.

Figure 2A is a truncated SEC trace of the NLP-rich peak after large scale assembly using the Superdex 200 26/60 column at a flow rate of 2.5 mL/min. Similar to the small scale assemblies, only a single NLP-rich peak was observed; however the NLP retention times in these experiments were different than the small scale assemblies (Figure 1A) due to the use of a different column. The vertical lines represent the fractions that were collected throughout the NLP-rich peak. NLP diameter distributions were determined by AFM and the relative fraction normalized to the SEC trace was calculated for each NLP size. Using this technique, the relationship between NLP size and elution time throughout the entire NLP-rich SEC peak was de-convoluted (Figure 2B). Interestingly, the elution of the four NLP sizes was not consistent across the entire peak. In fact, the maximal peak in the elution of larger particles occurred at earlier times where the peak elution times for the 28.0, 23.5, 19.0 and 14.5 nm NLPs were 49.9, 50.7, 51.5, 53.1 minutes, respectively (Figure 2B). Representative AFM images from three different fractions (highlighted in Figure 2A) are shown in Figure 2C. From these images it is clear that the size distributions vary throughout the NLP-rich peak.

**Figure 3.**
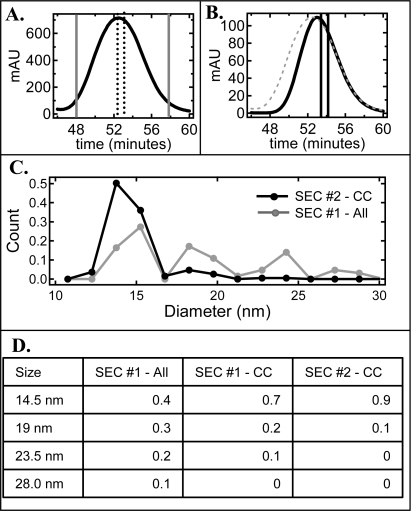
Center-cut SEC re-injection selectively purifies the smallest 14.5 nm NLPs. A. SEC trace of the first injection highlighting the NLP-rich peak (referred to as SEC #1). Dotted line indicates the center cut fraction that was subsequently re-injected (referred to as SEC #1 – CC). B. SEC trace of the second injection of the center cut material indicated in A., again highlighting the NLP-rich peak (referred to as SEC #2 - CC). Dashed SEC trace is the SEC #1 and black line represent the SEC fraction containing the most homogeneous NLP population (referred to as SEC #2 – CC). C. Histogram of diameter distributions for the pooled NLP peak from of SEC #1 (gray line) and the SEC #2 – CC fraction (black line). D. Table of the fraction of discrete NLP sizes (14.5, 19, 23.5, 28 nm) for three different SEC fractions, SEC #1 – All, SEC #1 - CC, and SEC #2 – CC.

Interestingly, the elution width of the 14.5 nm peak was narrower than that of the other sized particles and the majority of these particles eluted slightly to the right of the center of the entire NLP-rich peak (Figure 2B). Therefore, this region of the NLP-rich peak was highly enriched in the 14.5 nm size particle. In fact ~70% of the NLPs that eluted from 52.9 – 53.3 minutes were the ~ 14.5 nm sized NLP (referred to as SEC #1 –Center Cut), as opposed to ~ 40% for the pooled NLP-rich peak (referred to as SEC #1 – All). These results suggest that a second SEC injection of this elution fraction should result in a narrowing and shift in the main NLP peak. When this was done the peak in fact did narrow and shift to the right, indicating a more homogeneous sample of smaller particles (Figure 3B). To determine if there was an increase in NLP homogeneity in the center cut of the re-injected sample various elution fractions around this region of the NLP peak were imaged by AFM. Several fractions to the right of the center cut were found to be greater than 80% enriched in the 14.5 nm NLP but the fraction outlined by the black lines in Figure 3B (referred to as SEC #2 – Center Cut) was the fraction that contained the highest purity with 90% of the NLPs being ~ 14.5 nm in diameter (Figure 3C). The diameter distributions for the SEC #2 – Center Cut elution fraction and the SEC #1 – All elution fractions is given in Figure 3C. In addition, the exact fraction of the four different sized NLPs in the SEC #1 – All, SEC #1 – Center Cut, and SEC #2 – Center Cut elution fractions are given in Figure 3D.

These combined results indicate that the different sized particles are eluting as expected based on the principles of SEC but the presumed interactions severely tighten the difference in retention time; therefore, the different elution peaks of these particles appear as one single peak by SEC. Although the interactions severely hinder the ability of SEC to purify out a single NLP subpopulation it is possible to achieve homogeneity of the smallest 14.5 nm sized NLP through iterative SEC of the elution fraction that is to the right of the peak’s center.

### 2.3. Effect of homogeneity on NLP crystallization

One of the objectives of this study was to better understand the purification process of NLPs assembled using the cholate-free and cholate-associated methods in order to better produce homogeneous NLP sub-populations. The motivation for this approach is to utilize biophysical techniques that are sensitive to sample homogeneity. One such technique is the growth of high quality crystals for structural determination using X-ray crystallography. Obtaining discoidal lipoprotein crystals suitable for the determination of structure is a challenging task, although ellipsoidally-shaped full length apoE4 structures have been determined [30]. The benefits of these structures would significantly improve rational design of membrane protein solubilizing in NLPs. ApoE422k is of particular interest for solubilization of membrane proteins as it produces larger particles compared to other apolipoproteins [12] which may be relevant when accommodating large membrane proteins and protein complexes.

**Figure 4.**
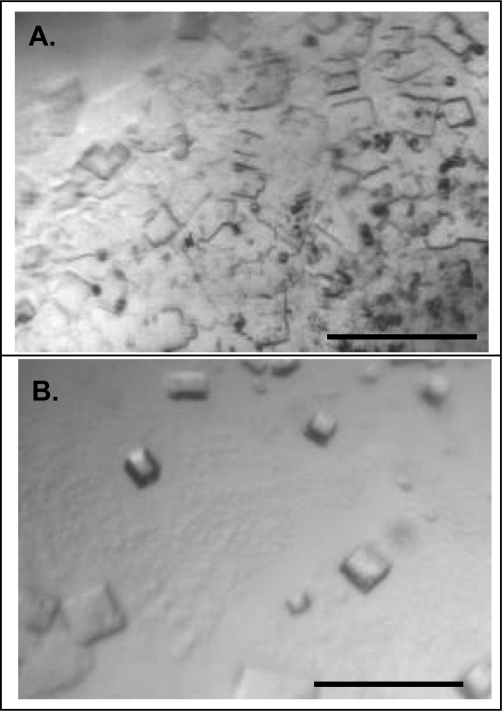
NLP crystal growth. A. Crystals grown from a polydisperse NLP sample (SEC #2 – CC). B. Crystals grown from a monodisperse NLP sample (SEC #1 – All). Scale bar 200 um.

To demonstrate the utility of a homogeneous and monodisperse NLP subpopulation, samples of varying homogeneity were subjected to *de novo* crystallization screening [31] and the quality of the resulting crystals were compared. For these studies, we chose to use NLPs assembled using the cholate-free method and side-by-side crystallization was carried out with NLPs pooled from the entire single NLP peak from the first SEC run (40% homogeneity) and NLPs from the two most homogeneous fractions from the second SEC run (90% homogeneity). Both samples showed a propensity to crystallize under similar conditions but the more homogeneous sample crystallized with higher propensity. However, there were notable differences in the crystallization habits of the two samples (Figure 4A and 4B). The less homogeneous sample tended to give smaller crystals that formed in the presence of an amorphous precipitate (Figure 4A) whereas the more homogeneous sample gave well formed crystals with less precipitate (Figure 4B). The best crystals, determined by crystal volume and clean morphology, were obtained from the more homogeneous sample. These results underline the benefit and importance of homogeneity for crystal growth. The largest crystals obtained to date from the highly homogeneous sample were ~100 μm in maximum extent and were birefringent. Optimization efforts are currently underway to obtain larger crystals.

## 3. Experimental Section

### 3.1. Materials

The phospholipid 1,2-dimyristoyl-sn-glycero-3-phosphocholine (DMPC) was purchased from Avanti Polar Lipids, Inc. (Alabaster, AL).

### 3.2. apoE422K protein production

The expression clone to produce apoE422K, the N-terminal 22 kDa fragment of apolipoprotein E4 (apoE4), as a 6His and thyrodoxin tagged construct was kindly provided by Dr. Karl Weisgraber. Production and purification of apoE422K has been described in detail elsewhere [12].

### 3.3. Nanolipoprotein particle (NLP) formation

NLP formation and purification both in the presence and absence of cholate have been described in detail elsewhere [12]. Briefly, dried DMPC was re-suspended in either 10 mM Tris pH 7.4, 0.15 M sodium chloride, 0.25 mM EDTA, 0.005% sodium azide (TBS) buffer followed by probe sonication to clarity (cholate-free assembly) or TBS/cholate (20 mM) (cholate associated assembly). ApoE422k (200–250 μg or 20 mg for large scale assembly) was added to the TBS/DMPC solution +/− cholate at a mass ratio of 4:1. The particle formation process was started with three repeated sets of transition temperature incubations, above (10 minutes at 30 °C, 20 minutes for large scale assembly) and below the transition temperature of DMPC (10 minutes at 20 °C, 20 minutes for large scale assembly) followed by incubation at 23.8 °C overnight (43 hours for large scale). Samples containing cholate were then dialyzed against 1000x volume of TBS buffer using 3 changes in 24 hrs. The NLPs were purified by size-exclusion chromatography using a Superdex 200 HR 10/300 column (GE Healthcare), in TBS at a flow rate of 0.5 mL/min or on a Superdex 200 26/60 at a flow rate of 2.5 mL/min for large scale preparations. The NLP fractions were concentrated to approximately 0.1 mg/mL using molecular weight sieve filters (Vivascience) with molecular weight cutoffs of 50 kDa. Protein concentration was determined using the ADV01 protein concentration kit (Cytoskeleton, Inc.).

### 3.4. Atomic force microscopy (AFM)

AFM images were acquired using a MFP-3D-CF AFM (Asylum Research, Santa Barbara, CA). Details of the imaging parameters have been described elsewhere [11,12].

### 3.5. Crystallization

Sets of 384 sitting-drop experiments were designed using our CrysTool crystallization design engine (Segelke, JCG 2001). Experiments were setup in IntelliPlate sitting drop trays using 100 μL of reservoir cocktail and 400:400 μL reservoir:NLP stock solution drops. NLP stock solutions were at a protein concentration of 2 mg/mL as determined by a Bradford assay. Both the highly homogeneous and less homogeneous samples showed a high propensity to crystallize in nearly the same set of conditions, where the more homogeneous sample crystallizing with slightly higher propensity. The lower homogeneity sample gives microcrystals or better in ~26% of CrysTool generated conditions and the higher homogeneity sample gives microcrystals or better in ~35% of the conditions tested. In addition, the lower homogeneity sample gave microcrystals or better in ~26% of CrysTool generated conditions and the higher homogeneity sample gives microcrystals or better in ~35% of the conditions tested. The large majority of conditions giving rise to NLP crystals are made up of mid molecular weight polyethylene glycol as the precipitating agent (2k-6k MW), a variety of buffer and range of pH (5.5–8.5).

## 4. Conclusions

In this study we found that NLPs assembled using the cholate-free method displayed a single peak and band by SEC and native gel, respectively, yet multiple sized particles were observed by AFM. In contrast, different sized NLPs, when the cholate-associated method was used, were effectively separated by SEC and native gel electrophoresis. Surprisingly, this phenomenon appears to be limited to E422k containing NLPs since different sized NLPs assembled with other apolipoproteins, such as apoA1, using cholate-free assembly methods have been previously reported to display multiple peaks and bands by SEC and native gel, respectively [15,16,25–28]. However, in light of the results of this study, one must be mindful of the techniques used to assess sample homogeneity since under some assembly conditions (i.e. the use of apoE422k and cholate-free assembly) neither SEC nor native gel electrophoresis has the sufficient ability to assess homogeneity. Therefore coupling these low resolution analytical separation techniques to high resolution image tools such as atomic force microscopy may be important so that heterogeneity can be appreciated in lipoprotein samples which appear homogeneous and also so that more homogeneous NLP assemblies can be obtained by appropriately fractionating SEC elution peaks. Additional analytical tools may be important as well to further understand the mass distribution of irregularly sized particles such as the NLPs, notably flow-field flow fractionation with multi-angle laser scattering detection (F4-MALS) [32,33]. We emphasize in this work that high resolution techniques can lead to a more rapid route to the generation of highly monodisperse self-assembled lipoproteins and crystal structures of them.

When chromatography with extremely short elution times was used to deconvolute the elution pattern of the different sized NLPs under cholate-free assembly conditions, we found that the elution of the four NLP sizes was not consistent across the entire single NLP peak. In addition, through iterative SEC of this peak, we were able to achieve 90% homogeneity of the 14.5 nm sized NLP. Although this is one pathway to produce a homogeneous NLP subpopulation under cholate-free assembly conditions this is by no means the only method. As was observed in the cholate-associated assembly ~ 80% of the NLPs in the first NLP peak were 23.5 nm in size. Therefore, it is likely that fine elution chromatography may be used in conjunction with cholate-associated assembly for the purification of a highly homogeneous NLP subpopulation of 23.5 nm particles. Finally, when the homogeneous NLP sample, in addition to a heterogeneous sample, were subjected to de novo crystallization we were able to demonstrate that the homogeneous sample produced much higher quality crystals than the heterogeneous sample; thus, opening up the potential for structural determination by X-ray crystallography.
